# Comparative analysis of gingival crevicular fluid and peri-implant crevicular fluid by mid-infrared spectroscopy: a split mouth study

**DOI:** 10.1007/s00784-025-06382-6

**Published:** 2025-05-20

**Authors:** Francisco Maligno, Ricardo N. M. J. Páscoa, Pedro S. Gomes

**Affiliations:** 1 Faculty of Dental Medicine, University of Porto, Porto, Portugal; 2 LAQV/REQUIMTE, Department of Chemical Sciences, Faculty of Pharmacy, University of Porto, Porto, Portugal; 3 BoneLab – Laboratory for Bone Metabolism and Regeneration – Faculty of Dental Medicine, University of Porto, Porto, Portugal; 4 LAQV/REQUIMTE, Faculty of Dental Medicine, University of Porto, Portugal Porto,

**Keywords:** Mid-infrared spectroscopy, Gingival crevicular fluid, Peri-implant crevicular fluid, Chemometric models

## Abstract

**Objectives:**

This proof-of-concept study aimed to compare the biochemical composition of gingival crevicular fluid (GCF) and peri-implant crevicular fluid (PICF) under healthy conditions, through mid-infrared (MIR) spectroscopy.

**Materials and methods:**

Using a split-mouth design, GCF and PICF samples were collected from 12 participants and analyzed through MIR spectroscopy. Advanced chemometric models, including partial least squares-discriminant analysis, k-nearest neighbors, and support vector machine discriminant analysis, were applied to explore potential biochemical differences between the biofluids.

**Results:**

No cluster formation was observed with PCA, indicating a high degree of similarity between groups. The PLS-DA model didn’t effectively discriminate between GCF and PICF with prediction rates of 62.5% (10/16) for calibration, 37.5% (6/16) for cross-validation, and 50% (4/8) for validation. The k-NN model, using k = 3 neighbors showed 25% (4/16) correct classification rates during calibration and a validation set accuracy of 50%. SVM-DA analysis showed a correct prediction rate of 37.5% (6/16) for calibration and 50% for cross-validation 50% (8/16) and 50% (4/8) in the validation phase. Nonetheless, subtle spectral differences were observed in spectral regions R1 (3982–2652 cm⁻^1^) and R4 (1180–922 cm⁻^1^), suggesting a slightly increased lipidic content and the presence of ethers and glycosidic bonds linked to carbohydrates, in PICF.

**Conclusions:**

The lack of significant biochemical differences between GCF and PICF under healthy conditions, as determined by MIR spectroscopy, suggests that implant-related changes in PICF composition are negligible.

**Clinical relevance:**

The demonstrated biochemical similarity between GCF and PICF under healthy conditions reinforces the potential of PICF as a reliable biofluid for diagnostic applications, including monitoring oral and systemic health biomarkers, without significant influence from implant-related factors.

## Introduction

Gingival crevicular fluid (GCF) is a serum-derived fluid found within the gingival sulcus, where it plays a crucial role in maintaining periodontal homeostasis. Under healthy conditions, GCF primarily functions as a transudate, formed by the passive diffusion of serum components through the junctional epithelium [1, 2]. It contains small amounts of proteins, cytokines, enzymes, growth factors, electrolytes, host-response modifiers, lipids, metabolites, tissue breakdown products, and bacterial byproducts [3]. However, in response to various stimuli – such as localized inflammation in gingivitis or periodontitis, or systemic conditions – the increased permeability of blood vessels transforms GCF into an exudate [4]. This shift is characterized by enhanced fluid flow and elevated levels of immune and inflammatory mediators [3, 5]. These dynamic changes makes GCF a valuable resource for monitoring biochemical changes associated with both oral and systemic health conditions [2, 5].

Similarly, peri-implant crevicular fluid (PICF) forms around dental implants, exhibiting functional similarities to GCF [1, 4, 6]. Structurally, the peri-implant environment resembles the dentoalveolar region of natural teeth, with key differences arising due to development variations. Notable distinction include the absence of periodontal ligament, reduced vascularity, and an increased presence of fibroblasts in peri-implant tissues [4, 6]. These structural differences are acknowledged to influence microbiota colonization, host immune responses, and tissue repair mechanisms [7–9]. As a result, while GCF and PICF share functional characteristics, their biochemical profiles may differ due to differences in tissue structure and cellular activity [1, 3, 4].

Fourier-transform infrared (FTIR) spectroscopy has emerged as a rapid, cost-effective technique for generating detailed chemical profiles of oral biofluids [10]. FTIR measures the absorption of infrared light by molecular bonds, providing a unique spectral fingerprint that reveals the presence of its molecular constituents, as proteins, lipids, nucleic acids, carbohydrates [11]. The mid-infrared (MIR) sub-region (within the 4000–400 cm^−1^ range) is particularly useful for analyzing organic species, making it ideal for identifying key functional groups in biofluids [12]. However, the acquisition of mid-infrared spectra requires the application of chemometric models to allow the extraction of meaningful information as the spectra are complex, due to weak and sometimes overlapped vibrations of different chemical bonds. FTIR’s non-invasive nature and analytical precision have made it valuable for the identification of both systemic and local conditions upon oral biofluids analysis. Systemically, FTIR has demonstrated potential in detecting infectious diseases, metabolic disorders, autoimmune conditions, and neoplastic processes [13–19]. Within the oral environment, it has been used to identify and monitor a range of oral disorders, including periodontal disease, oral cancers and mucosal conditions [14, 20–24]. Its high sensitivity to subtle biochemical changes supports its growing use in clinical research, with emerging developments in real-time diagnostics and personalized medicine.

Despite the established utility of FTIR spectroscopy in oral biofluid analysis, a critical gap remains in research directly comparing the spectral profiles of GCF and PICF under healthy conditions. Considering the structural and functional differences between periodontal and peri-implant tissues, it is plausible to hypothesize that GCF and PICF may possess unique biochemical signatures. Conducting a comparative analysis of these biofluids is essential to determine whether the presence of dental implants significantly alter the biochemical composition of PICF. Such an evaluation is critical not only to understand if implant-specific factors influence PICF but also to explore its potential as a viable alternative to GCF for diagnostic applications. Establishing a robust baseline of biochemical similarities and differences between GCF and PICF under healthy conditions will clarify whether PICF can reliably support diagnostic applications and biomarker discovery or if implant-related compositional changes might restrict its clinical utility.

This study aims to address this knowledge gap by conducting a proof-of-concept clinical assessment using a split-mouth design and MIR spectroscopy to evaluate the biochemical profiles of GCF and PICF. The study tests the null hypothesis that no significant biochemical differences exist between GCF and PICF under healthy conditions.

## Materials and methods

This study adhered to the Strengthening the Reporting of Observational studies in Epidemiology (STROBE) guidelines [25, 26], and incorporated relevant elements of the CONSORT extension for within-person trials to address the within-subject design [27]. Data collection took place between January 2023 and December 2024 at Dentalderme, a private dental clinic in Figueira da Foz, Portugal. The study protocol was approved by the Ethics Committee of the Faculty of Dental Medicine, University of Porto (FMDUP), under process number 000086. All participants provided written informed consent after receiving a thorough explanation of the study’s purpose, procedures, and potential risks.

### Study design and setting

This observational study employed a split-mouth design, in which GCF was collected from molar mandibular teeth in one quadrant, and PICF was collected from posterior mandibular implants in the opposite quadrant of the same participant. This design reduces the influence of confounding factors such as age, overall health status, oral hygiene habits, lifestyle, medications, ensuring the contribution of each participant to both groups.

Research design was developed to reduce potential bias forms. Selection bias was minimized through rigorous inclusion and exclusion criteria, ensuring homogeneity among participants and adherence to established guidelines. Bias due to inter-individual variability was reduced by adopting the split mouth study design and selecting participants with the same implant design and confirmed peri-implant health status. To reduce the information bias, sampling procedures were conducted similarly for both groups according to published protocols. To reduce investigator bias, standardized procedures were used for sample collection, as well as blinded data analysis. Confounding factors were managed by conducting detailed clinical histories and oral evaluations, ensuring clear inclusion or exclusion based on well-defined criteria.

### Participants

Twelve patients were screened between January 2023 and December 2024 by a single examiner (FM) using a structured questionnaire, medical history assessment, and clinical examination. Participants were selected based on the following inclusion criteria: adults aged between 20 and 90 years with the legal autonomy to provide written informed consent, the presence of at least one mandibular implant (NobelParallel Conical Connection, Nobel Biocare®) in functional loading for a minimum of 24 months and its contralateral natural tooth (posterior mandibular molar), and adherence to a regular maintenance therapy. Exclusion criteria included a history of periodontal or peri-implant disease (active or treated), pregnancy or breastfeeding, the presence of systemic, metabolic or immunological conditions, a history of smoking (current or past), or the use of medications such as nonsteroidal anti-inflammatory drugs, corticosteroids, immunosuppressants, or systemic antibacterial agents within 90 days prior to sampling.

**Fig. 1:**
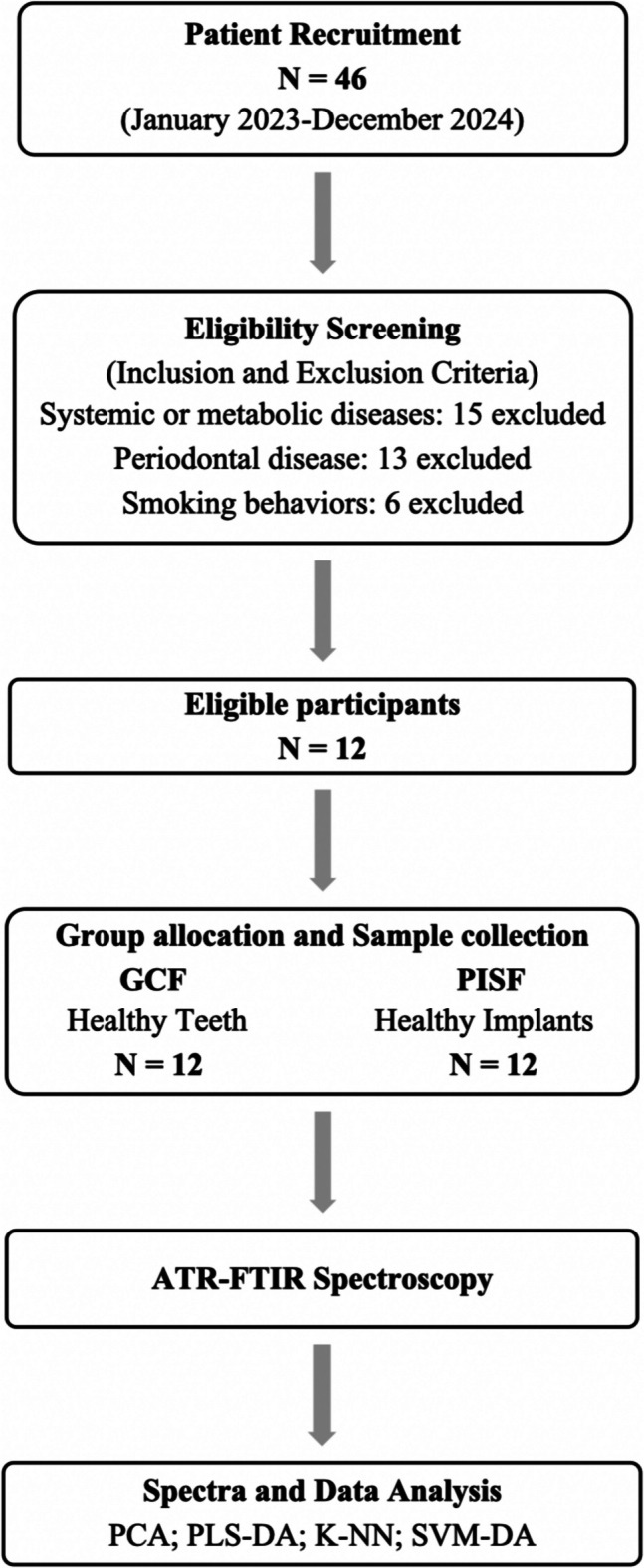
Flowchart illustrating patient recruitment, eligibility screening, group allocation (healthy gingival crevicular fluid vs healthy peri-implant crevicular fluid), sample collection and spectral data analysis

Following eligibility confirmation, PICF samples were collected from the posterior mandibular implants, with the peri-implant health diagnosis established according to the Consensus report of World Workshop on the Classification of Periodontal and Peri-Implant Diseases and Conditions [28]. GCF samples were collected from the contralateral posterior mandibular tooth, ensuring consistency in collection protocols across groups. All 12 participants contributed samples to both the GCF (*n* = 12) and PICF (*n* = 12) groups. The study flow is illustrated in Fig. 1, outlining the steps of participant recruitment, eligibility screening, group allocation, sample collection, and data analysis.

### Collection of oral biofluids

Oral biofluid samples of GCF and PICF were collected for each tooth and its contralateral implant using Periopaper® collection strips (ORAFLOW, Smithtown, NY, USA), following an established protocol [20, 22]. Prior to collection, the supragingival biofilm was carefully removed. The sampling sites were isolated with cotton rolls and the area was gently dried with controlled air flow. Collection strips were inserted in the mesiobuccal aspect of the gingival or peri-implant sulcus until light resistance was felt, and left in place for 30 s. The paper strips were immediately placed into sterile 1.5 mL Eppendorf tubes, labeled, and stored at −80ºC until analysis. Any samples obviously contaminated by saliva or blood were rejected.

### Acquisition of mid-infrared spectra

MIR spectra from each sample were acquired using a DTGS detector and a PIKE Technologies Gladi ATR accessory equipped in a PerkinElmer Spectrum BX FTIR Systems spectrophotometer (Waltham, USA). The crystal was cleaned with alcohol and the background calibration was performed in air prior to each spectra acquisition. Periopaper® collection strips were placed on the ATR crystal for spectral acquisition in diffused reflectance mode and compressed with a pressure of 150 N cm^−2^. Spectral data were collected between 4,000 to 600 cm^−1^, with 8 cm^−1^ of resolution. Three spectra were recorded per sample, and the mean spectrum was used for subsequent analysis.

### Spectra and data analysis

A range of chemometric models were applied to the data acquired in this study. Principal component analysis (PCA) was utilized to verify the formation of clusters and assist in detecting outliers. The identification of outliers was conducted using Hotelling’s T^2^ (weighed sum of squared scores) and Q residuals (sum of squared residuals) statistics. A sample was considered an outlier only if its score in both statistics exceeded their respective threshold lines simultaneously [29].

As PCA is an unsupervised model, partial least squares—discriminant analysis (PLS-DA) [30], support vector machines – discriminant analysis (SVM-DA) [31] and *k*-nearest neighbor (k-NN) [32] models were applied to develop discrimination/classification models. Before applying any of the chemometric models, the spectra were mean-centered. The entire spectral dataset was randomly divided into a calibration set (70% of samples, *n* = 16) and a validation set (30% of samples, *n* = 8) [22]. The calibration set was used for both calibration and cross-validation, while the validation set was used as an independent dataset for validation.

PLS-DA is a discrimination model that uses categorical *Y* data to determine class membership. It reduces data dimensionality into latent variables (LV) to mitigate computational complexity and overfitting. The models aim to maximize the covariance between spectral data (X) and class data (Y). Classification of an unknown sample into a specific group is achieved by calculating the probability of a sample belonging to each group, with the highest probability determining the classification [30, 33]. Although this model can handle slightly non-linear data, it is more effective for linear data. In this study, 1 to 3 LVs were tested, showing consistent results. Therefore, only results considering 2 LVs were presented. After calibration, validation samples were projected onto the calibration model to evaluate model accuracy.

Bearing in mind that PLD-SA is more effective for handling linear data, two additional models, namely SVM-DA and *K*-NN, which are effective for non-linear data were also tested. SVM-DA is a power classification model for both linear and non-linear data. It focuses on maximizing the margins of the hyperplane that separates data classes. When a linear separation is not possible, SVM-DA uses a kernel function to transform the data into a higher-dimensional space, enhancing separation capability [31]. A radial basis function was used as the kernel to handle non-linear data. Two tuning parameters, gamma (γ = 0.03) and the cost regularization parameter (ϲ = 31.6), were optimized using leave-one-out cross-validation. Validation samples were then projected onto the calibration model to evaluate the classification accuracy.

*K*-NN is a non-parametric classification model that categorizes samples based on the distance to its k-nearest neighbors in the multidimensional space. The choice of k neighbors is crucial, as too many or too few neighbors can lead to underfitting or overfitting. It does not require data training during calibration, simplifying the computational process [32]. In this analysis, the Euclidean distance metric was used, testing *k* values between 2 and 5. No significant differences were observed when using different values of *k* neighbors. As the performance remained consistent across different values, the results for k = 3 were reported. Following calibration, the model’s accuracy was evaluated using the validation set. *K*-NN was also used, alongside SVM-DA, because it is more efficient for small data sets and provides a more intuitive interpretation of the results than SVM-DA.

**Fig. 2:**
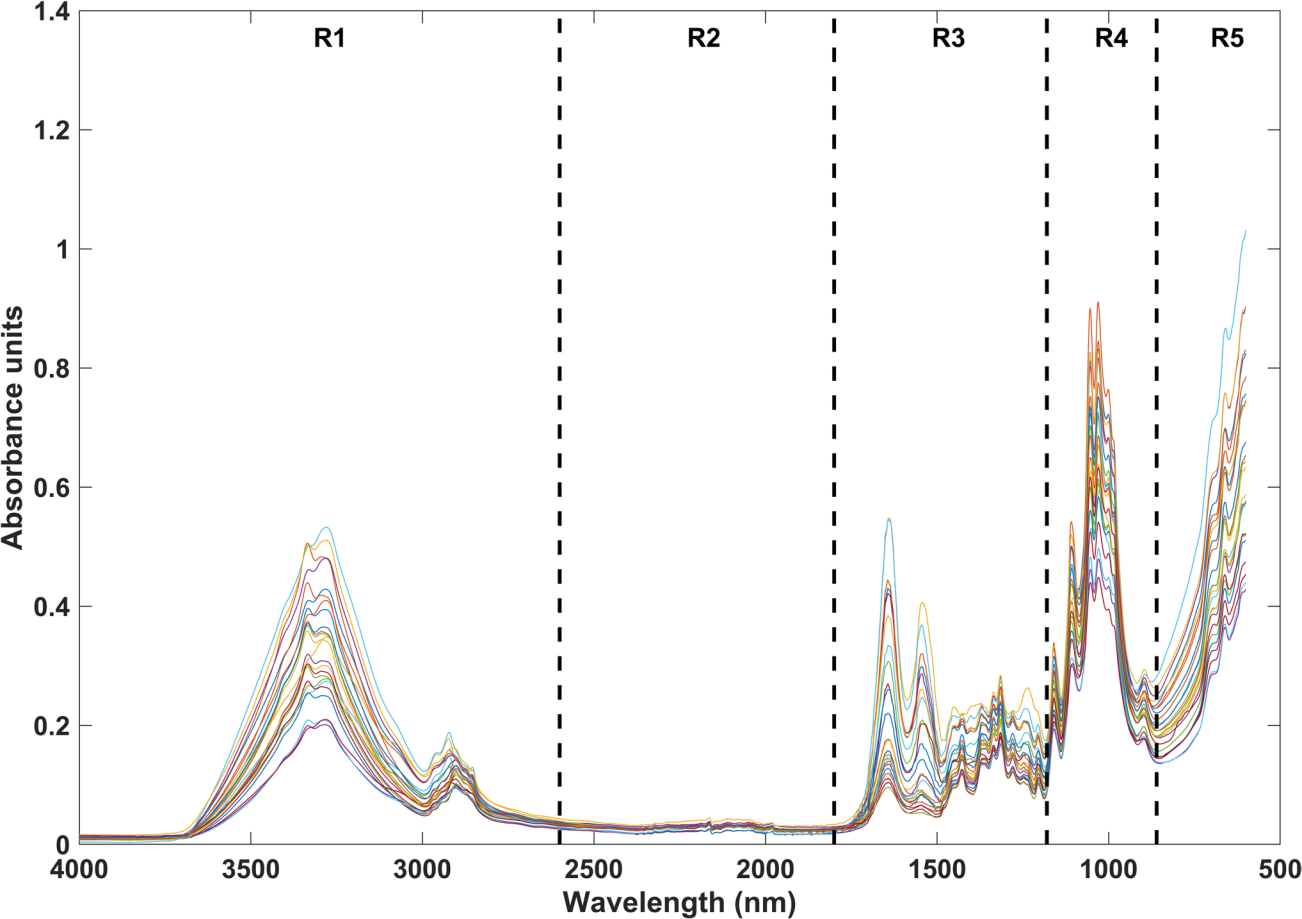
Raw MIR spectra of GCF and PICF samples collected from teeth and dental implant crevices, respectively. Five distinct spectral regions were identified and analyzed separately to assess differences between the two groups

MIR spectra were divided into five regions (Fig. 1) and tested individually and in combination. The spectral regions were established as follows: from 3982 to 2652 cm^−1^ (region 1), from 2650 to 1862 cm^−1^ (region 2), from 1860 to 1182 cm^−1^ (region 3), from 1180 to 922 cm^−1^ (region 4), and from 920 to 620 cm^−1^ (region 5). All spectral regions and preprocessing techniques were tested across the chemometric models. Since no significant differences were observed among the regions, the entire mean-centered spectral dataset was used for the final analysis (Fig. 2).

MIR spectroscopy generates a spectral image that reflects the superimposition of all infrared signals arising from the chemical constituents of the crevicular fluids, including proteins, lipids, nucleic acids, and carbohydrates.

The analysis was based on the Beer-Lambert law (Eq. 3), which correlates absorbance (*A*) with the concentration of the attenuating molecules:

3 $$A=\varepsilon\times\ell\times c$$

*A:* Absorbance.

*ε:* Molar absorption coefficient.

*ℓ:* Optical path length.

*c:* Concentration of the attenuating species.

The performance of the supervised models (PLS-DA, SVM-DA and *K*-NN) were assessed using confusion matrices. These matrices illustrated the overall accuracy of the models by summarizing the total percentage of correct predictions and providing sensitivity and specificity values. Specficity and were calculated using the following equations (Eq. 1 and 2), respectively:

1 $$Specificity=\frac{truenegativevalues}{truenegativevalues+flasepositivevalues}$$

2 $$Sensitivity=\frac{truepositivevalues}{truepositivevalues+falsenegativevalues}$$

All models and calculations were developed using Matlab R2023a version 9.14.0.2254940 (MathWorks, USA) using PLS Toolbox version 9.2.1 (Eigenvector Research Inc., USA).

## Results

From an initial pool of 46 participants, only 12 matched the eligible criteria after the initial screening. Participants were excluded due to systemic or metabolic diseases (15 individuals), history of periodontal disease (13 individuals), and smoking habits (6 individuals). The final sample consisted of 12 participants, each with one posterior mandibular tooth and a corresponding implant in the contralateral quadrant. The study sample included 7 woman (58.3%) and 5 men (41.7%), with a mean age of 61.7 years.

General characteristics of the participants, teeth, and implants studied are presented in Table 1.

**Table 1:** General characteristics of the participants, teeth, and implants studied

	Population (*N* = 12)	*p*-value
Age (years), mean (SD)	61.7 (11.5)	
Gender, N (%) Male Female	5 (41.7) 7 (58.3)	
Systemic disease N (%) No Yes	12 (100) 0 (0)	
Smoking behavior, N (%) No Yes	12 (100) 0 (0)	
Regular maintenance therapy, N (%) No Yes	0 (0) 12 (100)	
Periodontal disease history, N (%) No Yes	12 (100) 0 (0)	
OHI-S, mean (SD)	0.6 (0.3)	
Study group, N (%)		
Teeth (GCF)	12 (50)	
Implant (PICF)	12 (50)	
PD (mm), mean (SD)		< 0.001^a^
Teeth	1.99 (0.54)	
Implant	3.09 (0.98)	
BoP Index, mean (%)		0.777^b^
Teeth	13.89 (17.16)	
Implant	16.67 (30.98)	
Implant diameter (mm), mean (SD)	4.76 (0.34)	
Implants units, N (%)		
Single	7 (52.3)	
Part of bridge	5 (41.7)	

Total number varies according to missing data for each variable. Regular maintenance was defined as participating in an average of ≥ 1 supportive peri- implant care recalls per year. Abbreviations: *N* number, *SD* standard deviation. ^a^Student’s T-test. bWilcoxon test

### Preliminary analysis – raw data evaluation

**Fig. 3:**
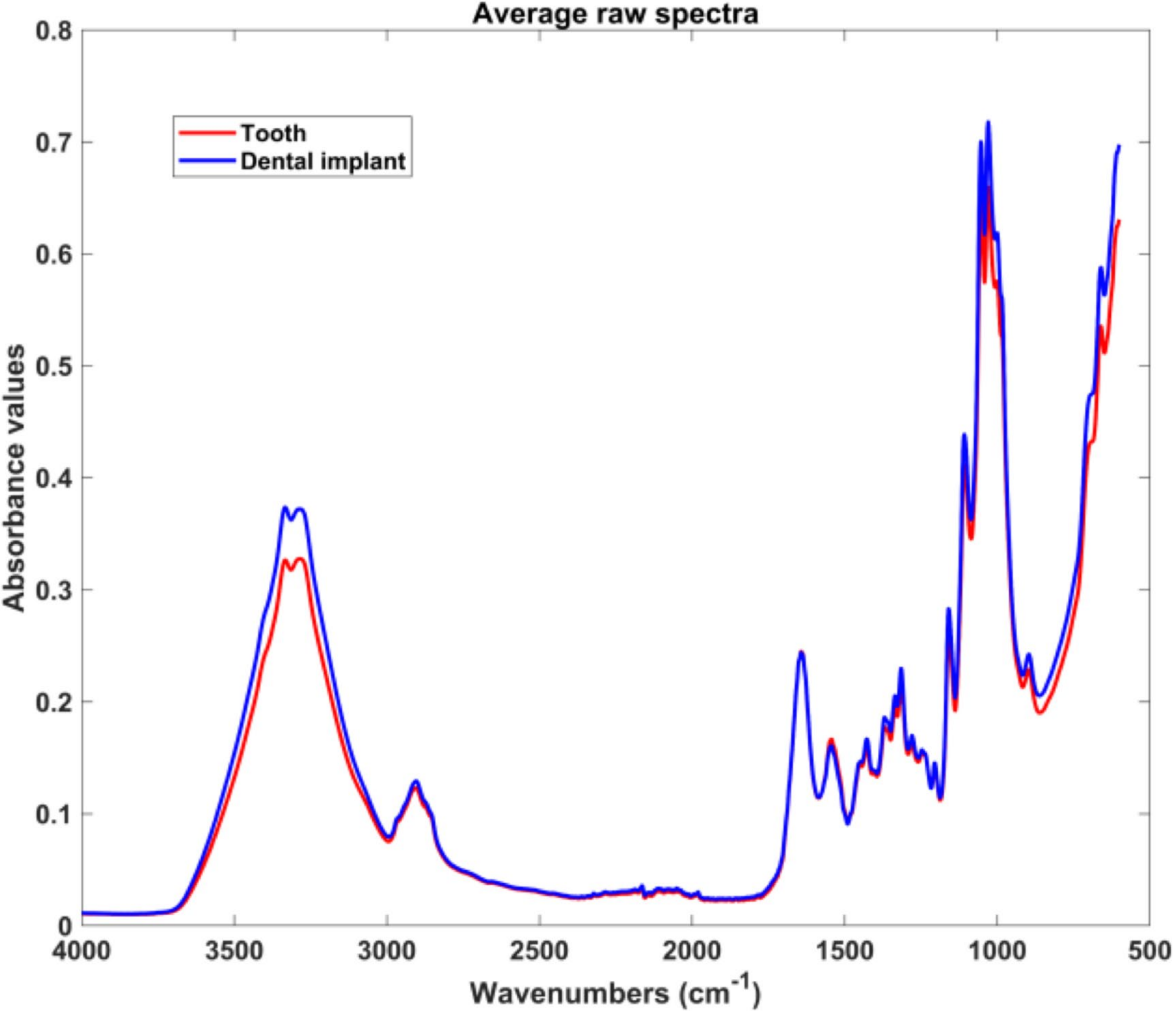
Average raw MIR spectra of GCF PICF samples collected from the crevices of teeth and dental implants, respectively

The raw and average MIR spectra for GCF and PICF samples are presented in Figs. 2 and 3, respectively. Key spectral features were identified in both GCF and PICF samples. Absorptions at 1652 cm⁻^1^ and 1542 cm⁻^1^ correspond to C = O stretching (amide I band) and N–H bending (amide II band) vibrations of peptide bonds, respectively. Additional spectral features included bands at 1087 and 1240 cm⁻^1^, which are associated with PO₂⁻ stretching vibrations of phosphodiester groups in DNA. The band at 1740 cm⁻^1^ is attributed to C = O stretching vibrations of lipid esters (14, 20). In the spectral range of 2800–3100 cm⁻^1^, stretching vibrations of CH₂ and CH₃ groups, primarily originating from lipids, were consistently detected in both groups. Further key spectral features included the amide B band at 3050 cm⁻^1^ and amide A band at 3290 cm⁻^1^, which are indicative of N–H stretching vibrations [14, 20].

While no statistically significant differences were observed between average raw spectra, PICF samples showed a slightly elevated amide II band peak, suggesting a higher average proteins or peptide concentration [14, 20]. Additionally, increased intensity of CH₂ and CH₃ stretching in PICF spectra pointed to a potentially higher lipid content in the peri-implant environment (20). Despite no distinct peaks or signal variations in the raw data, centered spectral analysis indicated higher peak intensities in spectral regions R1 (3982–2652 cm⁻^1^) and R4 (1180–922 cm⁻^1^) for PICF. These regions are associated with lipids and carbohydrates, suggesting a potential increase in lipidic profile and glycosidic bonds in PICF samples [14, 20].

### Principal component analysis

**Fig. 4:**
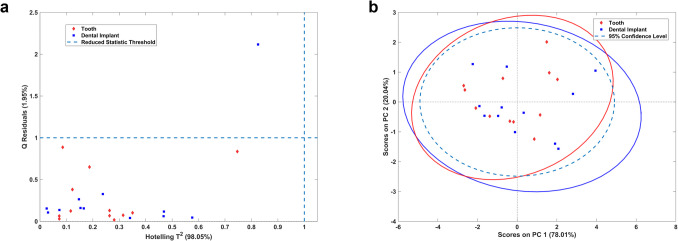
**a** Hotelling’s T^2^ plot (weighed sum of squared scores) and Q residuals (sum of squared residuals) statistics, evaluated using a 95% confidence interval, for outlier detection. **B** PCA scores plot of Principal Component 1 (PC1) *versus* Principal Component 2 (PC2), considering the entire spectra using 2 principal components. Spectra were previously mean centered

PCA was applied to verify the formation of clusters, as well as to assist in outlier detection (Fig. 4). No outliers were detected through the analysis of Hotelling’s T^2^ statistics and Q residuals, as observed in Fig. 3a, with all samples falling within the 95% confidence interval. PCA explained 98.05% of the total variance using the first two principal components. No distinct cluster formation was observed between the two sample groups, indicating a high degree of similarity in their MIR spectral profiles. These findings were consistent across all tested spectral regions and preprocessing techniques, suggesting the absence of significant biochemical differences between the groups.

### Supervised classification models: PLS-DA, SVM-DA and k-NN

The PLS-DA model produced correct prediction rates of 62.5% (10/16) for calibration, 37.5% (6/16) for cross-validation, and 50% (4/8) for validation (Table 2). The model did not effectively discriminate between GCF and PICF groups. Notably, in the validation set, all PICF samples were correctly classified whereas all GCF samples were misclassified. Specificity and sensitivity calculations further underscore this limitation. For the GCF group, specificity was 1 and sensitivity was 0, while for the PICF group, specificity was 0 and sensitivity was 1. These metrics underscored the complete misclassification of GCF samples and reinforced the model’s failure to distinguish between the two biofluids.

**Table 2:** Confusion matrices for the PLS-DA model applied to discriminate between GCF and PICF samples based on mean-centered MIR spectra, using the entire spectral range and employing two latent variables

Real	Calibration	
	GCF	PICF
GCF	7	1
PICF	5	3
	Cross-validation	
	GCF	PICF
GCF	3	5
PICF	5	3
	Validation	
	GCF	PICF
GCF	0	4
PICF	0	4

The k-NN model, using k = 3 neighbors and the entire mean-centered spectral dataset, showed a validation set accuracy of 50% (4/8) (Table 3). Consistent with PLS-DA results, all PICF samples were correctly classified while all GCF samples were misclassified. The validation set showed specificity and sensitivity values identical to the PLS-DA model results. GCF samples had a specificity of 1 and a sensitivity of 0, indicating complete misclassification. In contrast, PICF samples demonstrated a specificity of 0 and a sensitivity of 1, confirming perfect classification accuracy. During calibration, both groups demonstrated poor classification performance, achieving only 25% (4/16) correct classification rates. These results confirmed the model’s inability to distinguish patterns within the spectral data, highlighting the biochemical similarity of GCF and PICF.

**Table 3:** Confusion matrices for the *k*-NN model used to discriminate GCF and PICF samples based on mean-centered MIR spectra, considering the calibration and validation phases, using the entire spectral range and employing 3 k-neighbors

Real	Calibration	
	GCF	PICF
GCF	2	6
PICF	6	2
	Validation	
	GCF	PICF
GCF	0	4
PICF	0	4

SVM-DA analysis showed a correct prediction rate of 50% (4/8) in the validation phase (Table 4). Unlike the previous models, SVM-DA correctly classified GCF samples but misclassified all PICF samples. The confusion matrices showed varying classification patterns for calibration and cross-validation, but overall classification accuracy remained low, with 37.5% (6/16) for calibration and 50% for cross-validation (8/16). GCF samples had a sensitivity of 1 and specificity of 0, while PICF samples showed a sensitivity of 0 and specificity of 1, indicating complete misclassification.

**Table 4:** Confusion matrices for the SVM-DA model applied to discriminate between GCF and PICF samples, based on mean-centered MIR spectra of GCF and PICF, considering the calibration, cross-validation and validation phases using the entire spectral range

Real	Calibration	
	GCF	PICF
GCF	2	6
PICF	4	4
	Cross-validation	
	GCF	PICF
GCF	6	2
PICF	6	2
	Validation	
	GCF	PICF
GCF	4	0
PICF	4	0

## Discussion

Fourier-transform infrared (FTIR) spectroscopy has been applied to the analysis of oral biofluids due to its capability to provide detailed molecular fingerprints of biochemical composition [14–20, 22–24, 34–36]. Most studies using FTIR focus on detecting changes associated with inflammation, tissue degradation, and microbial activity, assisting in the diagnosis and monitoring of oral and systemic diseases [14–20, 22–24, 34, 35]. GCF and saliva are the primary biofluids examined in this context, with GCF offering insights into periodontal conditions and systemic diseases due to its serum-derived nature [18, 20, 22]. Saliva, due to its accessibility, is a versatile diagnostic medium for a wide range of conditions [10, 11, 13–17, 19, 21, 35, 37].

Peri-implant crevicular fluid (PICF) presents a promising yet underexplored biofluid for oral and systemic health monitoring. Unlike GCF, PICF has not been widely used, despite its potential to provide diagnostic insights from the peri-implant millieu, particularly concerning osseointegration and peri-implantitis [38, 39]. Beyond implant-related applications, PICF could also serve as an alternative to GCF for monitoring a broad range of oral [22] and systemic [40–43] biomarkers. However, the presence of a dental implant could significantly modify the biochemical composition of PICF [44]. These changes may arise directly from interactions with the metal-based biomaterial [45] of the implant or indirectly through implant-induced biological alterations [46], such as reduced vascularity, altered microbiota [44], and modulated host immune responses [46]. Such influences could either enhance or limit the clinical utility of PICF as a diagnostic substrate, necessitating a comparative study of GCF and PICF under healthy conditions to determine whether implant-induced compositional changes impact its diagnostic reliability.

In the present study, the analysis of MIR spectra from both GCF and PICF revealed no significant biochemical differences under healthy conditions, even after the application of various chemometric tools, including PCA, PLS-DA, k-NN, and SVM-DA. These findings support the null hypothesis that no significant biochemical differences exist between GCF and PICF under healthy conditions. The consistent lack of differentiation across all models suggests that PICF maintains a biochemical profile comparable to GCF, reinforcing its potential as a viable diagnostic biofluid for monitoring oral and systemic alterations. However, minor spectral variations were observed, such as slightly higher absorbance peaks in regions associated with lipidic content and glycosidic bonds (R1: 3982–2652 cm⁻^1^ and R4: 1180–922 cm⁻^1^) [13], being suggestive of subtle metabolic and tissue-specific responses to the peri-implant environment. These differences were not statistically significant and did not impact the overall chemometric comparison, suggesting that implant-related changes in PICF composition are minor under healthy conditions.

Several factors could account for the subtle variations in PICF composition. The peri-implant tissue environment has lower vascularity [4, 47], particularly in the connective tissue between the bone crest and the junctional epithelium [28]. This reduced vascularity may impair the transport and clearance of metabolites, such as lipids, contributing to their accumulation in PICF. Additionally, the absence of a periodontal ligament further reduces the metabolic clearance pathway around implants [48].

Additionally, the implants’ presence may subtly influence immune cell behavior [46, 49, 50]. With a typical composition of titanium (Ti) or zirconia alloys, and despite their biocompatibility and ability to integrate with surrounding tissues [51], the uniqueness of the surface topography and chemical properties of implants may modulate immune cell behavior, including the activation, recruitment and modulation of macrophages and neutrophils, even in the absence of pathological conditions [52]. Supporting this notion, previous data indicates a comparable cytokine profile between GCF and PICF under healthy conditions, albeit with minor differences in quantity [53, 54]. In addition, proteome analysis of GCF and PICF further supports our findings, demonstrating a strong similarity between these fluids under healthy conditions [55]. Their study revealed that only the"p53 pathway"was significantly enriched between teeth and implants, suggesting that differences in cellular metabolic regulation may exist, but are limited [55].

In addition, the release of ions or particles from implants, even at subclinical level, may subtly modulate immune activation and cytokine profiles in peri-implant tissues [56]. Microscopic wear, surface degradation, or corrosion may lead to the localized release of ions/particles [57], that may interact with immune cells, triggering the production of pro-inflammatory cytokines that could contribute eventual biochemical differences [58–60].

The distinct microbiota colonizing peri-implant environments may also contribute to the attained subtle differences between PICF and GCF. The structural and biochemical uniqueness of peri-implant tissues creates a microenvironment that influences the composition and behavior of the resident microbiota [61, 62]. This microbiota can directly influence epithelial cell function, modulate immune-inflammatory responses, and release specific mediators, such as lipopolysaccharides and proteases, that affect local cytokine production [63, 64].

The demonstrated biochemical similarity between GCF and PICF under healthy conditions, evidenced by the absence of significant differences in their MIR spectral profiles, underscores their shared role in maintaining tissue homeostasis. This finding supports the use of PICF as a reliable biofluid for diagnostic assessments, particularly since the presence of a dental implant does not appear to induce relevant compositional changes in PICF. The consistent spectral alignment between GCF and PICF reinforces the potential of PICF as a diagnostic tool not only for monitoring implant-related conditions such as peri-implantitis but also for broader clinical applications in assessing oral and systemic health biomarkers.

One of the limitations of this study is the exclusive focus on healthy sites. While our findings suggest that, in health, dental implants do not significantly alter PICF composition, this cannot be assumed for diseased states. While this approach was essential to establish a baseline comparison between GCF and PICF, it does not account for potential differences that may arise during pathological conditions such as periodontitis or periimplantitis. Structural and immunological variations between teeth and dental implants may exert a more pronounced influence on the biochemical composition of crevicular fluids during disease. Future studies should investigate GCF and PICF in diseased sites to better understand how these differences manifest under inflammatory conditions and to further evaluate the diagnostic capabilities of MIR spectroscopy in distinguishing periodontal from peri-implant diseases.

The overall findings suggest that, with further research and validation, PICF could become an integral component of clinical diagnostic protocols, particularly for monitoring peri-implant diseases such as mucositis and peri-implantitis. Additionally, due to its sensitivity to inflammatory and metabolic changes, PICF may hold potential for detecting systemic conditions with oral manifestations through the application of MIR spectroscopy.

## Data Availability

No datasets were generated or analysed during the current study.
